# Synergistic Efficacy of Doxycycline and Florfenicol Against *Aeromonas hydrophilia* and *Morganella morganii* Infections in *Pelodiscus sinensis* with Skin Ulcer Disease

**DOI:** 10.3390/vetsci12070611

**Published:** 2025-06-23

**Authors:** Ziwen Cai, Wenjing Zhang, Yun Wang, Zhaoying Yang, Xiaolei Lei, Xiaomin Shi, Mengze Du, Xiaoye Liu

**Affiliations:** Beijing Key Laboratory of Traditional Chinese Veterinary Medicine, Beijing University of Agriculture, No. 7 Beinong Road, Changping, Beijing 102206, China; caiziwen@bua.edu.cn (Z.C.); wenjingzhang@bua.edu.cn (W.Z.); graychief@163.com (Y.W.); m13581691358@163.com (Z.Y.); 18210078380@163.com (X.L.); shixiaomin@bua.edu.cn (X.S.)

**Keywords:** *Pelodiscus sinensis*, skin ulcer disease, antibiotic resistance, combination therapy

## Abstract

The intensive farming of *Pelodiscus sinensis* (*P. sinensis*) for its medicinal and economic value has led to an increased incidence of bacterial skin infections, commonly treated with antibiotics. However, rising antibiotic resistance has reduced the effectiveness of these treatments, highlighting an urgent need for alternative therapeutic approaches. In this study, we isolated and identified pathogenic bacteria (*Aeromonas* and *Morganella*) from *P. sinensis* affected by skin ulcer disease at a turtle farm in Beijing. Antibiotic resistance was evaluated using the checkerboard microdilution method and the in vitro antibacterial efficacy of a florfenicol–doxycycline combination therapy was assessed. A total of 30 bacterial isolates were obtained, primarily *Morganella* and *Aeromonas* species. Among these, 83.3% of *Morganella* and 57.14% of *Aeromonas* isolates demonstrated multidrug-resistance. The florfenicol–doxycycline combination yielded lower fractional inhibitory concentration (FIC) values, indicating synergistic or additive effects. Animal experiments have shown that florfenicol in combination with doxycycline can achieve better therapeutic effects with less drug. These findings support that combination antibiotic therapies help in managing skin ulcer disease in *P. sinensis* and offer valuable insights for clinical applications.

## Simple Summary

Skin ulcer disease has become a serious problem in Chinese soft-shelled turtle farming, leading to animal suffering and significant financial losses. The disease is caused mainly by bacterial infections, especially from *Morganella* and *Aeromonas* species, which have developed resistance to many commonly used antibiotics. This makes treatment increasingly difficult. In this study, bacteria were isolated from infected turtles and tested for their sensitivity to different antibiotics. We found that many strains showed resistance to multiple drugs. However, when doxycycline and florfenicol were used together, the combined treatment was much more effective than using either drug alone. This approach reduced the amount of antibiotics required, accelerated wound healing, decreased bacterial loads, and improved the survival rate of turtles. The combined therapy provides a practical solution to address drug-resistant infections in turtle aquaculture, helping to maintain animal health, prevent the spread of resistance, and support sustainable farming practices.

## 1. Introduction

Soft-shelled turtles (*Pelodiscus sinensis*, *P. sinensis*) are significant aquatic animals in China, holding substantial ecological and economic value. They serve dual purposes: as vital resources for the pet market and as an important food source. Global statistics indicate a steady increase in the production and farming of *P. sinensis* each year, particularly in regions like Asia and North America, where the turtle farming industry has become a major economic sector [1]. In China, turtle farming can be traced back to the 1990s, while in the United States, it dates back to the 1940s. Currently, China’s turtle aquaculture industry is renowned for its large-scale operations and serves as an integral part of the nation’s aquaculture sector, acting as a long-term driver of economic growth in farming regions. During the early development of the industry, various farming models emerged, such as courtyard farming, rooftop farming, and polyculture in fish ponds. These farming methods were typically loosely managed with relatively low stocking densities (0.75–1.5/m^2^). The rapid growth of turtle and soft-shelled turtle industry, particularly in response to the rising demand for turtle products in recent years, has led to a significant expansion of intensive farming practices (juvenile turtles: 20–30 turtles/m^2^; grow-out turtles (over 100 g): 15–20 turtles/m^2^; breeding turtles: 5–10 turtle/m^2^). However, this shift has also introduced new challenges. In high-density farming situations, pathogenic microorganisms can swiftly spread within farms, resulting in large-scale disease outbreaks that severely impact the economic viability for farmers [2]. Among these, skin ulcer disease is one of the most common and detrimental diseases encountered [3]. This situation underscores the importance of sustainable farming practices and effective disease management strategies to protect both the industry and *P. sinensis* themselves.

The farming of Chinese soft-shell turtles (*P. sinensis*) involves multiple stages. Juveniles are typically housed in plastic rearing tanks with water depth maintained at 1–2 times their shell height. Grow-out and broodstock turtles are generally cultured in ponds. While cement may occasionally be used for pond bottom waterproofing and hardening, this practice frequently causes plastron abrasions leading to infections. Earthen or sandy substrates are therefore strongly recommended. At all growth stages, abundant floating basking platforms must be provided. Water sources for turtle farming may include river water, rainwater, or groundwater, depending on local availability. Brackish water is rarely used except for marine turtles, though certain species like diamondback terrapins (*Malaclemys terrapin*) may require supplemental salt addition to water.

Skin ulcer is a common bacterial infection in *P. sinensis* that primarily affects their skin and shells. It often begins with limbs and abdomen, which can gradually develop into a moth-eaten appearance accompanied by white, decaying patches on the surface. In severe cases, discharge may emerge from the shell, usually accompanied by a foul odor, indicating that the infection has progressed significantly. This disease was first observed in high-density farming environments (10–15 turtle/m^2^) and has seen an increasing incidence in recent years due to the expansion of farming practices. Skin ulcer not only impacts the health and growth rates of *P. sinensis* but also leads to significant economic losses for farmers. The occurrence of skin ulcer is closely related to the farming environment [3]. *P. sinensis*, which predominantly live in aquatic settings, are particularly susceptible to factors such as water pollution, temperature fluctuations, and overcrowding, all of which can elevate the risk of infection [4]. Additionally, pathogens in turtle farming environments can spread rapidly through direct contact or contaminated water, resulting in widespread infections. In pathological studies of skin ulcer disease, scientists have identified several key pathogens responsible for this disease. Notably, Gram-negative bacteria are the primary culprits, including common species such as *Pseudomonas*, *Aeromonas*, *Morganella*, and *Shewanella*. Some Gram-positive bacteria, such as *Staphylococcus*, have also been associated with the onset of skin ulcer in *P. sinensis* [5,6,7,8,9]. These bacteria can enter the *P. sinensis* through wounds or contaminated water, proliferating and leading to localized or systemic infections [5,6,7,8,9]. Skin ulcer is an early-stage clinical manifestation in *P. sinensis*, along with other conditions such as scabies [10], blister [11], perforation [12] and red-neck disease [13]. In addition to bacteria, fungal pathogens like *Saprolegnia* and *Achlya*, viral agents such as iridoviruses, and parasites including mites and ciliates can also cause skin diseases in *P. sinensis* [14,15,16]. The diversity of pathogens complicates the clinical treatment of infectious skin diseases.

In recent years, the widespread use of antibiotics in turtle farming has increasingly highlighted the issue of antibiotic resistance among the pathogens responsible for skin ulcer. Antibiotic resistance refers to the ability of bacteria to develop resistance to antibiotics through mutations or horizontal gene transfer, rendering previously effective treatments ineffective. Pathogens causing diseases of aquatic animals have demonstrated varying degrees of resistance to commonly used antibiotics such as oxytetracycline, erythromycin, and sulfamethoxazole [17,18]. The emergence of this resistance not only significantly diminishes the effectiveness of single-antibiotic treatments for skin ulcer but also raises the risk of disease outbreaks and epidemics among *P. sinensis*. The development and spread of antibiotic resistance are directly linked to the inappropriate use of antibiotics. In turtle farming, improper treatment, preventive antibiotic use, and excessive reliance on these medications allow pathogens to adapt and evolve rapidly, resulting in the emergence of multidrug-resistant strains [19,20]. In China’s historical context, aquaculture medication decisions were predominantly made by farmers, and the discharge of antibiotic-laden effluent into natural water bodies accelerated the proliferation of resistant bacteria. However, recent regulatory mandates—including compulsory deployment of on-site veterinarians and stringent antibiotic usage protocols—are expected to markedly suppress antibiotic resistance emergence.

The presence of these resistant strains poses a dual threat: it impacts the aquaculture industry and may also endanger human health. The spread of multidrug-resistant bacteria limits treatment options and complicates management practices, consequently increasing the costs associated with farming.

Given the severity of the antibiotic resistance issue, the effectiveness of single antibiotics in treating skin ulcer has diminished significantly. In this context, the use of combination therapy with antibiotics has emerged as an effective strategy to combat bacterial resistance. By combining antibiotics with different mechanisms of action, combination therapy can enhance antimicrobial efficacy, reduce selective pressure for resistant bacteria, and inhibit the development of multidrug resistance [21,22]. The theoretical foundation for combination therapy lies in the synergistic effects between different antibiotics. When used together, they not only cover a broader spectrum of pathogens but also decrease the incidence of single-resistant strains [23,24]. Furthermore, combination therapy can lower the dosage of antibiotics required, thereby minimizing the potential toxicity to vital organs such as the liver and kidneys [25], ultimately enhancing treatment safety. Currently, combination therapy for treating skin ulcer in *P. sinensis* has shown promising results in both experimental studies and clinical treatments [26]. The concurrent use of antibiotics effectively controls the progression of skin ulcer and reduces the resistance of pathogenic bacteria.

In this study, we focused on isolating and identifying samples collected from a turtle farm in Beijing, specifically analyzing the most prevalent pathogens, *Aeromonas* and *Morganella* species, for antibiotic resistance. We propose a novel approach for treating skin ulcer disease using a combination of doxycycline and florfenicol. This combination not only broadens the spectrum of targeted pathogens but also mitigates the risk of developing resistance associated with single antibiotic use, thereby ensuring the treatment’s effectiveness. We aim for this new strategy to provide a more comprehensive solution for turtle health management and ultimately enhance farming efficiency. We hypothesize that one or more antibiotic combinations may effectively inhibit the growth of pathogenic bacteria causing skin ulcer disease in turtles, thereby achieving therapeutic efficacy with reduced antibiotic usage.

## 2. Materials and Methods

### 2.1. Sample Collection and Identification of Bacteria

Samples were collected from *P. sinensis* exhibiting clinical skin ulceration at a turtle farm in China between March 2023 and May 2023. A total of 55 specimens were obtained. All subjects presented characteristic signs of ulcerative dermatosis, including thickened, hemorrhagic lesions. During sampling, turtles were manually restrained by two operators, with procedures completed within five minutes.

Briefly, lesions were scraped using sterile blades, and material was suspended in 5 mL sterile PBS. Inocula were streaked onto blood agar plates using the quadrant method and incubated at 37 °C for 18 h. Single colonies were then transferred to 5 mL BHI broth and shaken at 220 rpm for 18 h. Putative isolates were identified through PCR amplification of 16S rRNA genes using primers 27F (5′-AGAGTTTGATCCTGGCTCAG-3′) and 1492R (5′-TACGGCTACCTTGTTACGACTT-3′) at 5 μmol/L concentration. Sequencing results were submitted to the National Center for Biotechnology Information (NCBI) database for BLAST 1.4.0 analysis against the GenBank database [27,28].

### 2.2. Antimicrobial Resistance Testing

Antimicrobial resistance testing of all the isolates were conducted using the broth microdilution method according to the Clinical and Laboratory Standards Institute (CLSI, 2020). *E. coil* ATCC25922 was used as the quality control strain. Antimicrobials commonly used in practice for treatment (cefotaxiofur sodium, enrofloxacin, meropenem, florfenicol, ampicillin, doxycycline, and hydrochloride) were selected for antimicrobial resistance testing.

Fractional inhibitory concentration index (FICI) was determined by checkerboard assays as described previously [29]. First, 100 µL MHB medium was dispensed into a 96-well plate. Doxycycline (100 µL) was added to the last row, and then diluted along the ordinate. Subsequently, florfenicol was added to the first column and diluted along the abscissa. Finally, in addition to the negative control, 100 µL bacterial suspensions ≈ 1 × 10^6^ CFUs mL^−1^ were added. After culturing at 37 °C for 18 h, the synergistic effect was determined by calculating FICI. All experiments were performed with at least six replicates. The FICI was calculated according to the following formula:

FICI = (MIC_ab_)/(MIC_a_) + (MIC_ab_)/(MIC_b_) = FIC_a_ + FIC_b_

where MIC_a_ and MIC_b_ are the minimum inhibitory concentrations of drugs a and b when used individually, and MIC_ab_ is the MIC when both drugs are used together. FIC_a_ is the FIC of compound a; FIC_b_ is the FIC of compound b.

### 2.3. Ethical Statement

The breeding and experimental operations of all experimental animals were carried out in strict compliance with the Guidelines for Laboratory Animal Use and Care from the Chinese Center for Disease Control and Prevention and the Rules for Medical Laboratory Animals from the Chinese Ministry of Health, under protocol BUA612503001, approved by the Animal Ethics Committee of Beijing University of Agriculture.

### 2.4. In Vivo Infection

*P. sinensis* is the most widely farmed turtle species in China for both culinary and medicinal purposes. It is highly accessible and clinically prone to ulcerative skin disease, making it an ideal model for infection experiments. In this study, according to the results of MIC in the previous stage (the FICI values were compared with the same drug resistance), *Morganella* strain 27 and *Aeromonas* strain 13 were the most resistant to antibiotics involved in this study, so these strains were selected as the challengers to verify the therapeutic effect of the combination in vivo. *Aeromonas* and *Morganella* were cultured for 24 h, and bacterial CFU were measured via the plate counting method before seeding [30].

Healthy *P. sinensis* (male and female, 8 weeks old, body weight = 15 ± 2 g) were randomly divided into 8 groups with 8 turtles per group: negative control, infected control (*Aeromonas* + *Morganella*), florfenicol-treated group (*Aeromonas*+ *Morganella*), doxycycline-treated group (*Aeromonas* + *Morganella*), dual antibiotic treatment group, and povidone–iodine (PVP-I)-treated group. PVP-I was previously reported as effective in the treatment of ulcer skin diseases.

Substances such as methylene blue, formaldehyde, and malachite green were also previously utilized but are prohibited now. Therefore, in our in vivo infection experiment, we selected PVP-I as the positive control. This choice aligns with efforts to promote scientifically standardized drug use in aquaculture, prevent excessive veterinary drug residues in aquatic products, and enhance their quality and safety. Supporting this goal, in 2022, the Ministry of Agriculture of the PRC consolidated regulations on aquaculture drugs, specifically recognizing doxycycline and florfenicol for treating aquatic animal diseases. This official recognition underscores the importance of discussing the use of these antibiotics, particularly in the current context of serious bacterial resistance.

For bacterial infection, the skin on the plastron of turtles was lightly scald with a 5 × 5 mm soldering iron (180 °C) and then immersed in water (2 cm) containing *Aeromonas* (1 × 10^5^ CFU/mL) and *Morganella* (1 × 10^5^ CFU/mL) for 24 h, respectively. Prior to the procedure, turtles were anesthetized using alfaxan (10 mg/kg intramuscularly, Zoetis). Then, 5 mg/L of PVP-I or 0.64 mg/mL of doxycycline or 0.64 mg/mL of florfenicol or both of them (0.32 mg/mL doxycycline + 0.32 mg/mL florfenicol) was added into the cultural aquatic environment, respectively. The control group underwent the same process but was immersed in sterile water. The negative control group did not undergo scald treatment and was kept in sterile water.

Every 5 days, skin lesions were swabbed using a cotton tip applicator (with a tip area of 20 mm^2^), and bacterial loads were assessed on a Rimler–Shotts selective medium agar (RS agar) for computing *Aeromonas* and blood agar plates for *Morganella* (the isolated *Morganella* strain exhibited hemolytic activity; consequently, we only count hemolytic colonies). Sampling occurred seven times over a 30-day period, and photographic documentation was taken throughout the study. On day 30, all animals were euthanized (the animals were anesthetized with isoflurane and euthanized by mandatory decapitation) [31], and skin samples from the shell were collected, fixed in 4% paraformaldehyde for 24 h, rinsed in running water for 12 h, and dehydrated in an ethanol gradient. The samples were embedded in paraffin, sectioned into 4 μm slices, and stained with hematoxylin–eosin (H&E) for histological analysis under a light microscope (Olympus, Tokyo, Japan).

To validate the identity of *Aeromonas* and *Morganella* in our in vivo experiment, samples from the infection control group were cultured on the RS agar medium and blood plates. On day 15, 20 colonies were picked up for 16S rRNA gene sequencing and BLAST comparison to validate the identity of the pathogenic bacteria as the inoculated strain, thus fulfilling the requirements of Koch’s postulates. The results of 16S rRNA sequencing showed that 18 bacteria on the isolated RS agar plates were aligned to *Aeromonas* and 19 bacteria on the blood plates were *Morganella*, indicating that the method used in this study to calculate the wound bacterial load was feasible.

### 2.5. Statistics

All plot graphs show the means ± sd. Statistical analysis for each independent experiment was performed with the Kruskal–Wallis test. *p* value of <0.05 was considered significant: * *p* < 0.05; NS, not significant (Graphpad Prism 9.5.1).

## 3. Results

### 3.1. Isolation and Identification of Bacteria

**Figure 1:**
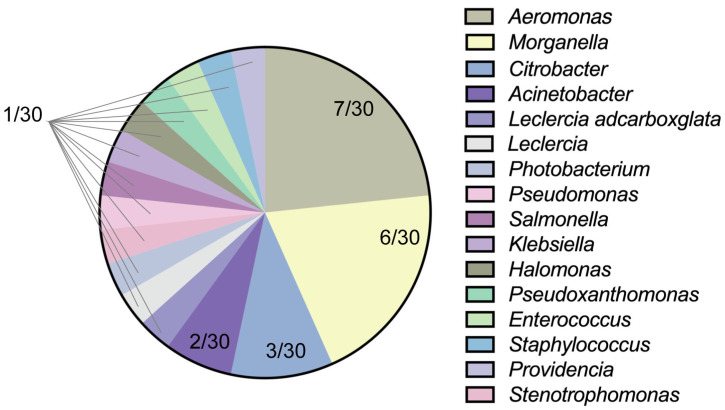
Bacterial isolation rate from skin ulcer disease in *P. sinensis*. *Aeromonas* and *Morganella* were the most prevalent.

In order to identify the pathogen that causes skin ulcer disease, we isolated and identified the bacteria in the collected samples. A total of 30 strains of bacteria were isolated from the collected samples, with 16 genera of bacteria: Gram-positive bacteria (7.14%, *n* = 2) and Gram-negative bacteria (92.86%, *n* = 28) (Figure 1). *Morganella* and *Aeromonas* were predominant with a detection rate of 20.00% (*n* = 6) and 23.33% (*n* = 7), respectively. Other genera included *Citrobacter* at 10.00% (*n* = 3), *Acinetobacter* at 6.67% (*n* = 2), and *Providencia*, *Leclercia, Achromobacter*, *Photobacterium*, *Stenotrophomonas*, *Pseudomonas*, *Salmonella*, *Klebsiella*, *Halomonas*, *Pseudoxanthomonas*, *Enterococcus*, and *Staphylococcus* at 3.33% (*n* = 1).

### 3.2. Molecular Identification and Hemolysis Analysis

**Figure 2:**
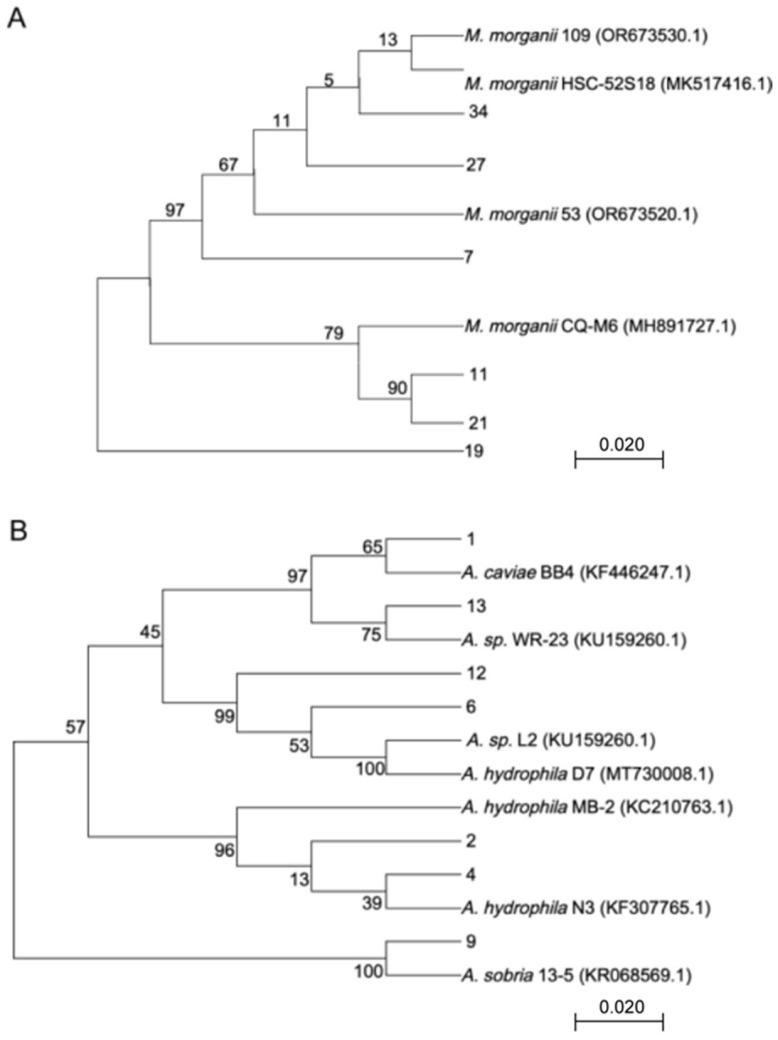
Phylogenetic trees of *Morganella* (**A**) and *Aeromonas* (**B**). Phylogenetic network generated using the neighbor-net algorithm with unselected P distances (bar = 0.020).

The primary pathogenic bacteria, *Morganella* and *Aeromonas*, were subjected to 16S rRNA sequence alignment and a phylogenetic tree was constructed, as shown in Figure 2. The similarity between the isolated *Morganella* strains and reference strains ranged from 89.64% to 99.70%, while the similarity of *Aeromonas* strains ranged from 86.67% to 99.12%, as detailed in Table 1. Hemolysis experiments were performed on the 13 isolated strains, revealing a hemolysis rate of 50% (*n* = 3) for *Morganella* and 28.57% (*n* = 2) for *Aeromonas*.

### 3.3. Antibiotic Resistance Analysis

After successfully isolating the target bacteria, we further evaluated their resistance to six major classes of antibiotics—chloramphenicols, cephalosporins, tetracyclines, β-lactams, carbapenems, and fluoroquinolones—to determine their minimum inhibitory concentration (MIC). The MIC values of *Morganella* for ceftiofur ranged from 64 to >128 μg/mL, doxycycline from 32 to >128 μg/mL, ampicillin from 128 to >128 μg/mL, enrofloxacin from 1 to 64 μg/mL, florfenicol at >128 μg/mL, and meropenem from 16 to >128 μg/mL. For *Aeromonas*, the MIC values for ceftiofur and ampicillin were >128 μg/mL, doxycycline ranged from 8 to >128 μg/mL, enrofloxacin ranged from 1 to >4 μg/mL, meropenem from 16 to >128 μg/mL, and florfenicol ranged from 128 to >128 μg/mL (Table S1).

The bacterial sensitivity varied across different antibiotic classes. Enrofloxacin exhibited the highest sensitivity, while resistance rates were 100% for chloramphenicols, cephalosporins, tetracyclines, β-lactams, and carbapenems, with a 69.23% resistance rate to enrofloxacin (Table 2). Additionally, *Morganella* showed more severe antibiotic resistance than *Aeromonas*, with 83.3% (*n* = 5) of isolates identified as multidrug-resistant strains, compared to 57.14% (*n* = 4) of *Aeromonas* isolates.

### 3.4. Combination Therapy

After determining the MIC of individual antibiotics against the bacteria, we further investigated the antibacterial effects of combining different classes of antibiotics on multidrug-resistant *Aeromonas* strain 7 to identify more effective treatment options. The results showed a synergistic effect with an FICI value of 0.375 when florfenicol was used in combination (Table 3, Figure S1). Next, to further confirm the effect of this combination, we analyzed eight isolated multidrug-resistant strains. The results of the combination therapy sensitivity study showed that the fractional inhibitory concentration index (FICI) values were all less than 1 when doxycycline and florfenicol were used together, indicating synergistic or additive effects.

**Figure 3:**
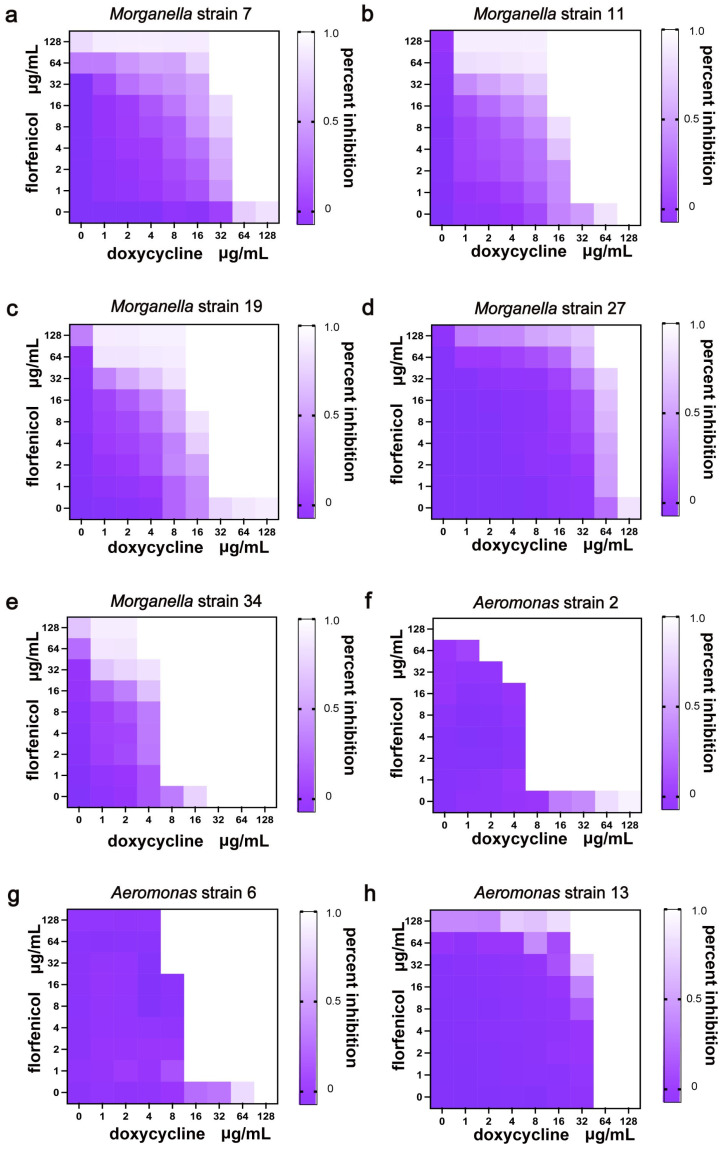
Antibacterial effect of combined doxycycline and florfenicol treatment on multidrug-resistant *Morganella* and *Aeromonas* strains. (**a**–**h**) Images correspond to bacterial isolates numbered 7, 11, 19, 27, 34, 2, 6, and 13, respectively.

Moreover, combination therapy can reduce the required drug dosage. For *Morganella*, the MIC of doxycycline decreased by 1–3 times, and the MIC of florfenicol decreased by 2–5 times. For *Aeromonas*, the MIC of doxycycline decreased by 3–6 times, and the MIC of florfenicol decreased by 2–3 times (Figure 3, Table 4).

Therefore, we recommend the clinical use of combined therapy with doxycycline and florfenicol for the treatment of multidrug-resistant *Morganella* and *Aeromonas* infections.

### 3.5. Combination Therapy on Infection Model

**Figure 4:**
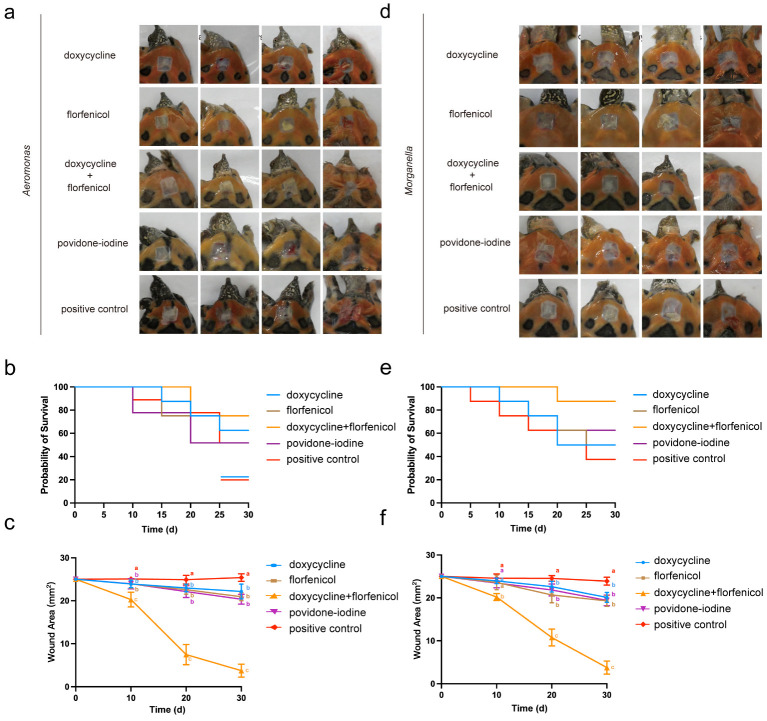
Therapeutic effects of doxycycline, florfenicol, and their combination on skin ulcers in turtles infected with *Aeromonas* (**a**–**c**) and *Morganella* (**d**–**f**). (**a**,**d**) Representative images of *P. sinensis* with skin ulcer disease in each treatment group. Groups include doxycycline, florfenicol, doxycycline + florfenicol combination, povidone–iodine, and positive control. (**b**,**e**) The Kaplan–Meier survival curves of *P. sinensis* in each treatment group during the 30-day observation period. (**c**,**f**) Changes in wound area (mm^2^) at days 0, 10, 20, and 30. Data are presented as mean ± standard deviation. Different letters indicate statistically significant differences between groups (*p* < 0.05).

We can see that the wound healing rate of the turtles in the combined drug group was much faster than that of the single drug group and the iodophor group from the fifth day onwards. From the photos, we can intuitively see that the combined drug group shrinks the wound quickly, and we can also see that the symptoms of redness and edema of the lesion are significantly better than those in the other groups (Figure 4a–c), especially when compared to the control group. From the mortality curve, we can see the deaths in the double antibiotic group on day 20, while the PVP-I treatment and control groups began to die on day 10, and in the single antibiotic group on day 15 (Figure 4b).

According to Figure 4d–f, we can see that after *Morganella* infection, the wound recovery rate was the fastest in the double antibiotic treatment group, and there was a significant difference between the other groups on the fifth day. From Figure 4e, we can see similar results to the *Aeromonas* group, where only one individual died 20 days after infection, and the time of death was later than that of the other groups.

**Figure 5:**
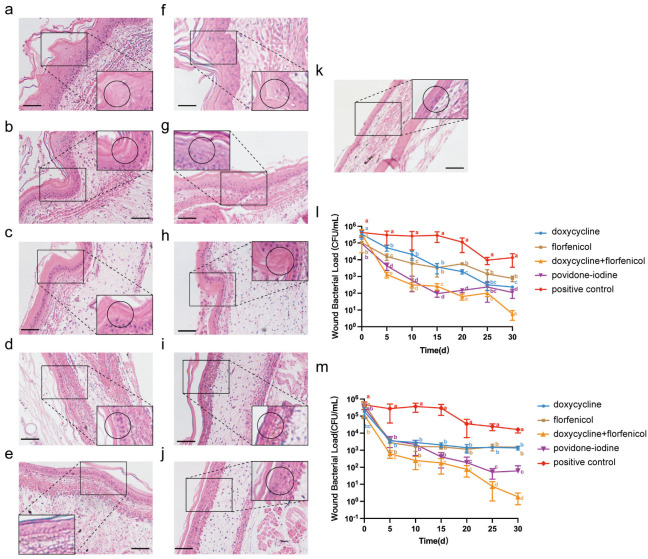
Histological analysis and bacterial load in skin lesions of turtles treated with different therapies. (**a**–**j**) Representative H&E-stained histological sections showing the epidermal and dermal layers of skin lesions from untreated and treated turtles (bar = 100 μm). (**k**) Negative control. (**l**,**m**) Bacterial load in the wound (CFU/mL) over 30 days of treatment, measured at different time points. Data are presented as mean ± standard deviation. Different letters indicate statistically significant differences between groups (*p* < 0.05).

As shown in Figure 5, thickened stratum corneum and loose keratin were seen in the epidermal layer of the skin of the untreated turtle, and the presence of keratinocytes that had not yet been enucleated. The number of keratinized cell layers is also increased, and up to 5–6 layers can be seen. Edema may be seen in the dermis with the presence of a large number of inflammatory cells. The combination group compared with the untreated control group, the thickness of the stratum corneum in the epidermis was thinner, the number of keratinized cell layers were only 3–4, and the degree of edema in the dermis was also lower. In contrast, the combination group showed fewer layers of epithelial cells and a denser and thinner keratinotic layer in the epidermis, and the edema and inflammation of the dermis were similar to those in the group alone.

Our data on the bacterial load in the wound are consistent with the pathological data from the animal experiments described above. The bacterial load on the wound in the bispecific antibody group was much lower than in the untreated group and was significantly lower at 15 days compared to the Figure 5d,f groups.

## 4. Discussion

To gain a deeper understanding of the bacterial infections causing skin ulcer disease in *P. sinensis*, this study isolated and identified bacteria from skin ulcer lesions. 16S rDNA sequences were aligned, and a phylogenetic tree was constructed. A total of 16 bacterial genera were identified, with 92.86% being Gram-negative bacteria. Among them, *Morganella* and *Aeromonas* were predominant, accounting for 21.43% and 25%, respectively. Other identified genera included *Acinetobacter* and *Citrobacter.* Research indicates that several other key pathogenic bacteria are also associated with turtle skin ulcer disease, such as *Aromatoleum* [32], *Pseudomonas fluorescens* [33], and *Alcaligenes* [34].

Currently, antibiotics remain the primary treatment for bacterial skin ulcer disease in *P. sinensis.* However, the widespread use of antibiotics in aquaculture has led to increasing bacterial resistance. This study evaluated the antibiotic resistance of the collected *Morganella* and *Aeromonas* isolates. The results showed that 83.3% of *Morganella* isolates were multidrug-resistant, while 42.86% of *Aeromonas* isolates exhibited multidrug resistance. The lowest resistance rate was observed for enrofloxacin (69.23%). Previous studies have demonstrated that enrofloxacin antibiotics are effective against over 90% of *Aeromonas* strains [35], suggesting that enrofloxacin can be recommended as the first-choice antibiotic for treating turtle skin ulcer disease. However, 4-quinolones are banned for use in China.

However, antibiotic resistance varies significantly due to differences in farming environments and pathogens. For instance, Liu et al. reported that *Aeromonas*-induced skin ulcer disease in *P. sinensis* responded well to ceftazidime, cefoperazone, cefotaxime, gentamicin, amikacin, kanamycin, netilmicin, levofloxacin, ofloxacin, and enrofloxacin [36]. In this study, the antimicrobial effect of florfenicol, ceftiofur, doxycycline, ampicillin, and meropenem antibiotics was weak (100% resistance), indicating that the problem of antimicrobial resistance has become more serious in turtle and turtle culture in China. Given the potential risks associated with antibiotic resistance, it is crucial to explore alternative treatment strategies.

Given the serious issue of antibiotic resistance and mixed infections in bacterial skin ulcer disease in *P. sinensis*, combination antibiotic therapy has become a research focus. This approach can reduce drug dosages, slow the development of resistance, and broaden the spectrum of treatment. In this study, the combined use of doxycycline and florfenicol not only lowered the required dosage but also enhanced the antibacterial effect.

When used alone against *Morganella*, the MIC of doxycycline ranged from 32 to 128 μg/mL, and the MIC of florfenicol was >128 μg/mL, indicating high resistance. However, with combination therapy, the MIC of doxycycline decreased to 4 to 64 μg/mL and that of florfenicol decreased to 8 to >128 μg/mL. For *Aeromonas*, the MIC of doxycycline alone ranged from 8 to >128 μg/mL and florfenicol alone had an MIC of 128 to >128 μg/mL. With the combination treatment, the FICI values for both bacteria were less than 1, indicating synergistic or additive effects. Florfenicol acts as a bacterial 50S ribosomal subunit inhibitor while doxycycline targets the 30S subunit, and their synergistic interaction provides dual blockade of bacterial protein synthesis. However, in this experiment, we observed significant strain-dependent variability in the combined effects of doxycycline and florfenicol. This phenomenon may be attributed to the presence of antibiotic efflux pumps or other resistance genes in certain strains that counteract the synergistic action. Our results clearly demonstrate this resistance pattern, which will be the subject of further investigation in subsequent studies.

In addition to combination antibiotic therapy, several other strategies are available for controlling drug-resistant bacteria. These include treatment with plant-derived natural compounds, antibody–antibiotic conjugates (AACs) [37], antimicrobial peptides (AMPs) [38], specific antibodies [39], and bacteriophages. However, research on turtle skin ulcer disease remains limited, with most studies focusing on pathological analysis and identification of pathogenic bacteria, leaving significant gaps in understanding.

According to the approved veterinary drugs for aquaculture marked on the aquaculture medicines published by the Ministry of Agriculture and Rural Affairs in 2022, we found that florfenicol and doxycycline can be used in aquaculture in China. In terms of therapeutic effect, the bispecific antibody treatment group showed better treatment effect in terms of histopathological convenience, wound bacterial load, and wound recovery speed [40,41]. Therefore, future research should emphasize the infection mechanisms of skin ulcer disease and explore more effective treatment strategies to address this issue comprehensively.

There are several limitations of our study. First, for the isolated pathogenic strains, we did not conduct in-depth pathogenicity comparisons or screen for resistant gene and virulence factors, which significantly limited their reference value. Additionally, our sampling scope was relatively restricted; future studies should expand the diversity of sample sources. Although this trial explored an antibiotic-reduction treatment protocol, non-antibiotic therapeutic routes represent the critical research direction for scientific aquaculture. Emerging alternatives—such as plant extracts and animal-derived antimicrobial peptides (AMPs) [42,43]—have demonstrated potent bacteriostatic efficacy. Consequently, these approaches should be prioritized in research agendas and accelerated toward commercial application.

## 5. Conclusions

This study demonstrated that *Morganella* and *Aeromonas* are the primary pathogens responsible for skin ulcer disease in *P. sinensis*. Despite the high MIC values of doxycycline and florfenicol, the combination therapy of these antibiotics showed significant therapeutic efficacy. By combining these two antibiotics, the required drug dosages were substantially reduced, and synergistic or additive effects were observed, especially against multidrug-resistant strains of *Aeromonas* and *Morganella.*

The use of this combination therapy not only reduced the total amount of antibiotics used but also enhanced the antimicrobial activity against these resistant pathogens. This approach has important implications for the management of antibiotic resistance, as it slows the development of resistance and broadens the spectrum of treatment. Furthermore, this strategy is significant for aquatic animal health, particularly in intensive aquaculture settings. By reducing the reliance on high-dose antibiotics, this approach helps prevent the spread of antibiotic-resistant strains and improves the overall health of farmed aquatic animals, contributing to the sustainability of aquaculture practices.

Future research should further investigate the mechanisms behind this combined treatment, analyze the variability of resistance patterns in different strains, and explore the potential of non-antibiotic therapies such as plant-derived compounds and animal-derived antimicrobial peptides to combat the growing issue of antibiotic resistance.

## Figures and Tables

**Table 1:** 16S rRNA similarity of *Morganella* and *Aeromonas*.

Code	Reference Strain	Similarity
7	*P. morganii* (MK517416.1)	99.30%
11	*P. morganii* (MH891727.1)	89.64%
19	*P. morganii* (OR673530.1)	99.70%
21	*P. morganii* (MH891727.1)	99.40%
27	*P. morganii* (OR673520.1)	99.40%
34	*P. morganii* (OR673520.1)	99.70%
1	*A. caviae* (KF446247.1)	95.39%
2	*A. hydrophila* (KC210763.1)	99.12%
4	*A. hydrophila* (KF307765.1)	97.31%
6	*A. hydrophila* (MT730008.1)	97.21%
9	*A. sobria* (KR068569.1)	86.67%
12	*A.* sp (HQ292718.2)	97.21%
13	*A.* sp (KU159260.1)	98.45%

**Table 2:** Statistics of resistance rates of different types of antibiotics.

Category	Antibacterial Agents	Rate (%)		
		R	I	S
Chloramphenicols	Florfenicol	100.0	0	0
Cephalosporins	Ceftiofur	100.0	0	0
Tetracyclines	Doxycycline	100.0	0	0
β-lactams	Ampicillin	100.0	0	0
Carbapenem	Meropenem	100.0	0	0
Fluoroquinolones	Enrofloxacin	53.5	46.15	0

**Table 3:** FIC values for combined antibiotic therapies with strain 7.

Drug Name	MIC (μg/mL)		Drug Name	MIC (μg/mL)		FIC	Combined Results
	Single	Combined		Single	Combined		
Ceftiofur	>128	128	Meropenem	>128	128	2	Synergistic
		>128	Enrofloxacin	128	128	2	Synergistic
		>128	Ampicillin	>128	>128	2	Synergistic
		1	Doxycycline	128	128	1.008	Synergistic
Florfenicol	>128	64	Enrofloxacin	128	64	0.75	Additive
		16	Doxycycline	128	32	0.375	Synergy
		128	Ceftiofur	>128	4	0.516	Additive
		>128	Ampicillin	>128	>128	2	Synergistic
		128	Meropenem	>128	1	0.516	Additive
Ampicillin	>128	128	Enrofloxacin	128	32	0.75	Additive
		128	Doxycycline	128	128	1.5	Synergistic
		128	Meropenem	>128	128	1	Additive
Meropenem	>128	32	Doxycycline	128	64	0.625	Additive
Enrofloxacin	128	64	Meropenem	>128	1	0.504	Additive
		64	Doxycycline	128	2	0.516	Additive

**Table 4:** FIC index of doxycycline and florfenicol against multidrug-resistant *Morganella* and *Aeromonas* strains.

Strain	Doxycycline MIC (μg/mL)		Florfenicol MIC (μg/mL)		FICI	Combined Results
	Single	Combined	Single	Combined		
7	128	32	16	>128	0.375	Synergistic
11	128	16	>128	16	0.1875	Synergistic
19	128	16	>128	16	0.1875	Synergistic
27	128	64	>128	64	0.75	Additive
34	32	8	>128	32	0.375	Synergistic
2	>128	4	128	32	0.2656	Synergistic
6	128	8	>128	32	0.1875	Synergistic
13	>128	32	>128	32	0.25	Synergistic

## Data Availability

The datasets presented and generated in this work are available on request to the corresponding author.

## Supplementary Materials

The following supporting information can be downloaded at https://www.mdpi.com/article/10.3390/vetsci12070611/s1. Figure S1: Heat map of different antibiotics combined. Table S1: Determination of minimum inhibitory concentrations for *Morganella* and *Aeromonas*.
