# Supplementary material for: Synergistic Efficacy of Doxycycline and Florfenicol Against Aeromonas hydrophilia and Morganella morganii Infections in Pelodiscus sinensis with Skin Ulcer Disease
Source: Vet Sci. 2025 Jun 23;12(7):611. doi: 10.3390/vetsci12070611 (PMC12298653; doi:10.3390/vetsci12070611)

Table S1: Determination of minimum inhibitory concentrations for *Morganecia* and *Aeromonas*

Species	Code	MIC（μg/mL)					
		CEF	DOX	AMP	ENR	MEM	FFC
Morganella	7	>128	128	>128	4	128	>128
	11	>128	128	>128	8	>128	>128
	19	>128	64	>128	8	>128	>128
	21	>128	64	128	1	16	>128
	27	64	128	>128	16	64	>128
	34	>128	32	>128	64	128	>128
Aeromonas	1	>128	8	32	1	16	>128
	2	>128	>128	>128	2	32	128
	4	>128	8	>128	4	16	>128
	6	>128	128	>128	2	>128	>128
	9	>128	64	>128	1	128	>128`
	12	>128	64	>128	1	128	>128
	13	>128	128	>128	2	64	>128
Acinetobacter	5	-	-	-	-	-	-
	8	-	-	-	-	-	-
Citrobacter	10	-	-	-	-	-	-
	14	-	-	-	-	-	-
	15	-	-	-	-	-	-
Leclercia adcarboxglata	16	-	-	-	-	-	-
Achromobacter	17	-	-	-	-	-	-
Photobacterium	18	-	-	-	-	-	-
Stenotrophomonas	20	-	-	-	-	-	-
Pseudomonas	21	-	-	-	-	-	-
Salmonella	23	-	-	-	-	-	-
Klebsiella	24	-	-	-	-	-	-
Halomonas	26	-	-	-	-	-	-
Pseudoxanthomonas	28	-	-	-	-	-	-

Figure S1:Heat map of different antibiotics combined.

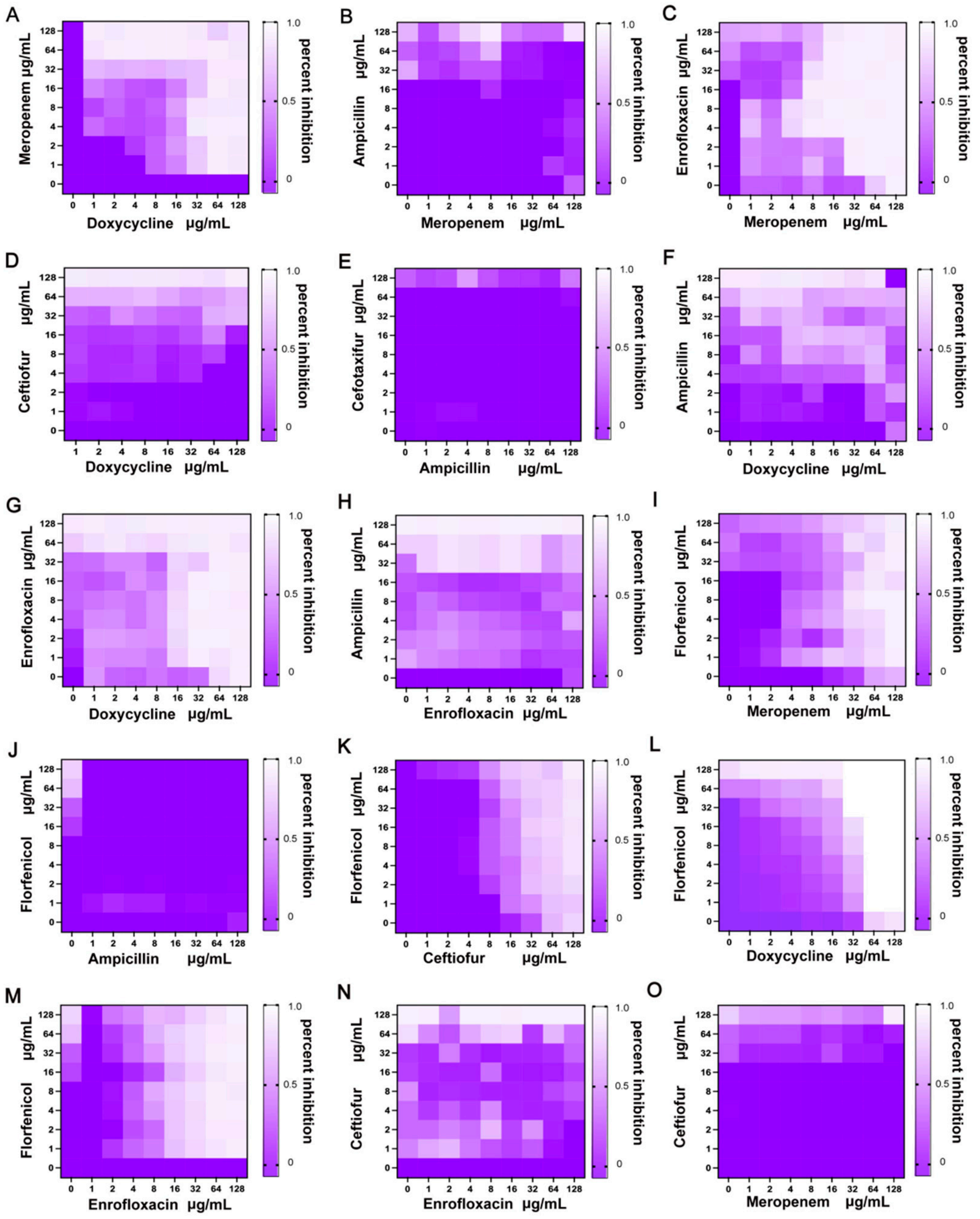

Supplement: Supplementary file 1 [file vetsci-12-00611-s001.zip › vetsci-3642058-supplementary.pdf]
